# Visualization of the Left Extraperitoneal Space and Spatial Relationships to Its Related Spaces by the Visible Human Project

**DOI:** 10.1371/journal.pone.0027166

**Published:** 2011-11-07

**Authors:** Haotong Xu, Xiaoxiao Li, Zhengzhi Zhang, Mingguo Qiu, Qiwen Mu, Yi Wu, Liwen Tan, Shaoxiang Zhang, Xiaoming Zhang

**Affiliations:** 1 Department of Anatomy, College of Basic Medical Sciences, Third Military Medical University, Chongqing, People's Republic of China; 2 Department of Radiology, Nanchong Central Hospital of North Sichuan Medical College, Nanchong, Sichuan, People's Republic of China; 3 Department of Health Service, College of High Altitude Military Medicine, Third Military Medical University, Chongqing, People's Republic of China; 4 College of Bioengineering and Medical Imaging, Third Military Medical University, Chongqing, People's Republic of China; 5 Center for Advanced Imaging Research, Medical University of South Carolina, Charleston, South Carolina, United States of America; 6 Sichuan Key Laboratory of Medical Imaging, Department of Radiology, Affiliated Hospital of North Sichuan Medical College, Nanchong, Sichuan, People's Republic of China; University of Illinois at Champaign-Urbana, United States of America

## Abstract

**Background:**

The major hindrance to multidetector CT imaging of the left extraperitoneal space (LES), and the detailed spatial relationships to its related spaces, is that there is no obvious density difference between them. Traditional gross anatomy and thick-slice sectional anatomy imagery are also insufficient to show the anatomic features of this narrow space in three-dimensions (3D). To overcome these obstacles, we used a new method to visualize the anatomic features of the LES and its spatial associations with related spaces, in random sections and in 3D.

**Methods:**

In conjunction with Mimics® and Amira® software, we used thin-slice cross-sectional images of the upper abdomen, retrieved from the Chinese and American Visible Human dataset and the Chinese Virtual Human dataset, to display anatomic features of the LES and spatial relationships of the LES to its related spaces, especially the gastric bare area. The anatomic location of the LES was presented on 3D sections reconstructed from CVH2 images and CT images.

**Principal Findings:**

What calls for special attention of our results is the LES consists of the left sub-diaphragmatic fat space and gastric bare area. The appearance of the fat pad at the cardiac notch contributes to converting the shape of the anteroexternal surface of the LES from triangular to trapezoidal. Moreover, the LES is adjacent to the lesser omentum and the hepatic bare area in the anterointernal and right rear direction, respectively.

**Conclusion:**

The LES and its related spaces were imaged in 3D using visualization technique for the first time. This technique is a promising new method for exploring detailed communication relationships among other abdominal spaces, and will promote research on the dynamic extension of abdominal diseases, such as acute pancreatitis and intra-abdominal carcinomatosis.

## Introduction

The LES is a potential space in the sub-diaphragmatic area. It represents a thin fat strip between the diaphragm and the posterior wall of the proximal portion of the stomach, the lesser curvature on CT or MRI images. There is no obvious density difference between the LES and its adjacent spaces. Detailed anatomic features of the LES are not prone to be displayed perfectly on normal cross-sectional CT scans or multiplane reconstruction (MPR) images. By contrast, the thin-slice cross-sections derived from the Chinese and American Visible Human Project (CVHP, VHPA) and the Virtual Human dataset of Bethune Medical College (VHBM) are brightly colored, the images are clear, and histological structures are satisfactorily displayed [1]–[3]. Furthermore, the cross-sections from these datasets, other random sections, and the 3D digital models obtained allow visualization of complicated anatomic structures, such as the renal fascia and abdominal spaces. The 3D visualization technique has also been used in studying organs, tissues, and in previous small animal studies [4]–[8]. Here, we use the visualization technique to study the anatomic features of the LES.

The Materialise's Interactive Medical Image Control System (Mimics®) software is an interactive tool for visualization and segmentation of gray-value images and 3D rendering of objects. The Amira® software has the advantage of showing the colorful images of Visible Humans as well as CT images on random sections. Therefore, both types of software were used in the current study.

The location and morphology of the anteroexternal surface and internal structures of the LES have been explored by gross anatomy and thick-slice sectional anatomy [9]–[11]. However, there are some controversial issues regarding the spatial relationship between the LES and the gastric bare area (GBA). Conventionally, most anatomists, radiologists, and surgeons identified the LES with the GBA, and used GBA terminology to refer to the LES [9], [10], [12], [13]. But Min and his colleagues argued in 2003 that parts of the left sub-diaphragmatic fat had participated in composing the LES except for the GBA [14]. The lack of agreement over the spatial relationship between the LES and GBA leads to difficulties in academic communication about various diseases involving the LES. Furthermore, in anatomical and radiological research, opinions differ regarding the morphology of the anteroexternal surface of the LES, which also creates problems. Most researchers regard it as an inverted triangle [9], [12], [15], [16], although Chen et al. (2001) and Lu et al. (2009) viewed it as an upright trapezoid [17], [18]. Using 3D visualization, the detailed spatial relationships of the LES to its related spaces, especially to the GBA, can be clearly displayed, and the morphology of the anteroexternal surface of the LES can be made clear.

The associated vessels of the LES can be involved in severe acute pancreatitis, and the lymph nodes within the LES can be invaded in gastric carcinoma. In acute pancreatitis, LES involvement has a characteristic CT finding and could serve as a useful prognostic indicator for the disease [12]. In cases involving proximal gastric carcinoma, the LES was often invaded [16]. Due to the invasion of the LES, cancers of the posterior wall of the proximal stomach were more difficult to completely resect than were those of the anterior wall [16], [19], [20]. As an important sub-diaphragmatic space during the pathological progress of severe acute pancreatitis and gastric carcinoma, we should place a high value on the anatomical study of the LES.

Our study investigated the spatial relationships of the LES to its related spaces, especially the GBA, and should help clarify the morphology of the anteroexternal surface of the LES based on the CVHP, VHPA, and VHBM datasets. This may be helpful for diagnosis of LES abnormalities, and for preoperative planning, as well as simulation of various procedures for treating vascular disorders that involve the LES.

## Materials and Methods

### 1 Ethics statement

The study was approved by the Ethics Review Board of the Third Military Medical University. The First and Second Chinese Visible Human datasets (CVH1, CVH2) of the CVHP were from a voluntary donation [21], [22]. Written consent was obtained from relatives of the participants in CVH1 and CVH2. CT scans in five volunteers were approved by the Institutional Review Board of the Southwest Hospital of the Third Military Medical University. Written informed consent was obtained from the five volunteers before the CT scans, and no identifiable information (i.e. age and gender) was used in this study.

### 2 3D reconstruction of the LES based on CVH2

#### Cross-sectional data of the upper abdomen from the CVHP

The CVH1 and CVH2 datasets were from a 35-year-old man and a 22-year-old woman, respectively. Both cadavers were without organic disease and were donated on a voluntary basis. The successive thin-slice cross-sectional images of the upper abdomen with 170 mm thickness, from the dome of the diaphragm to the lower pole of the left kidney, were retrieved from the CVH2 dataset for 3D reconstruction. For both cadavers, preliminary CT and MR scans were performed to exclude lesions of the upper abdomen before milling. The cadavers were placed in a supine position, with the median sagittal plane of the upper abdomen parallel to the body's long axis. The slice interval was 0.5 mm and the resolution was 6,291,456 (3,072×2,048) pixels. Each TIFF file occupied 36 Mb (approximate pixel size 167 µm) as previously described [21], [22].

#### Segmentation

With Photoshop CS® (Adobe Systems Incorporated, USA), the images were registered through four reserved fiducial rods and the background was removed. In addition, the structures including the LES, left gastric artery and vein, posterior gastric vein, left gastric lymph nodes, left cardiac lymph nodes, abdominal portion of the esophagus, stomach, and pancreas were outlined with the magnetic lasso tool, and the outlines of different structures were filled with distinct colors using the “fill” command. After changing the 16-bit color to 2-bit color, color-filled images were transferred to gray-scale images. The 340 gray-scale images retrieved from CVH2, including the above structures without background, were imported to Mimics V14.0® (Materialise Company, Belgium) and a new stack file with the expanded name “mcs” was created. With the “Thresholding” tool provided by Mimics V14.0®, the above structures were segmented on a threshold basis, and each structure was labeled with a different mask.

#### Superficial outline reconstruction

**Figure 1:**
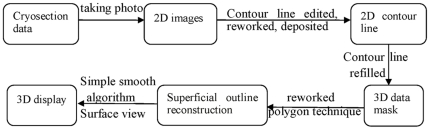
Flow chart of visualization of the LES and its related structures.

After segmentation, the superficial outline reconstruction command in the 3D objects menu was used to reconstruct the 3D LES of CVH2 and its internal and related structures. This reconstruction method was based on polygon technology. The surfaces of 3D-reconstructed structures were smoothed with the smooth surface command. Fig. 1 shows the detailed flow chart of visualization of the LES and its related structures. The models of superficial outline reconstruction of the LES and its internal structures, and the abdominal portion of esophagus, stomach, and pancreas were then exported from Mimics V14.0® to Amira 5.2.0® (TGS Company, Australia) with a ply format for displaying.

#### MPR images of the upper abdomen

The above 340 serial cross-sectional images of the upper abdomen from the CVH2 dataset were imported to Amira® to obtain the orthoslices or obliqueslices of the upper abdomen via MPR. The orthoslice or obliqueslice images were observed in a successive manner, clearly and directly [4].

### 3 Anatomy of the LES and spatial relationships to related spaces

In addition to the CVH1 and CVH2 datasets, the Virtual Human dataset of Norman Bethune Medical College was provided by Jilin University [2], [8]. The digital dataset for Visible Human male of American (VHMA) was obtained from the US National Library of Medicine (NLM) [1], [23]. The anatomical location of the LES and the morphology of its anteroexternal surface were explored on consecutive cross-sections of the CVH1, CVH2, VHBM, and VHMA, and the 3D models of CVH2. The spatial relationships of the LES to the GBA, lesser omentum, hepatic bare area, and left retroperitoneal space, and the distribution of the internal structures of the LES were also investigated in the above four datasets.

### 4 LES on CVH2 images versus MSCT images

Five volunteers (four male and one female; mean age 36.4 years, range 24–54) were recruited to undergo contrast-enhanced 64-slice spiral CT scans (General Healthcare, Milwaukee, WI, USA). All volunteers were healthy and without history of abdominal surgery. 150 ml iodine contrast medium was injected (300 mg/ml) through any available superficial vein in the cubital fossa, and images were transmitted to Amira® software. The upper abdomen multidetector CT (MSCT) coronal and oblique sagittal images were reconstructed using MPR technique. The reconstructed thickness of MPR was 0.33 mm and the reconstructed interval was 0.33 mm. The anatomical location of the LES and its spatial relationships to the hepatic bare area, lesser omentum, and left retroperitoneal space on MSCT images were studied and compared with that from CVH2 images.

## Results

### 1 Anatomy of the LES

#### Location

**Figure 2:**
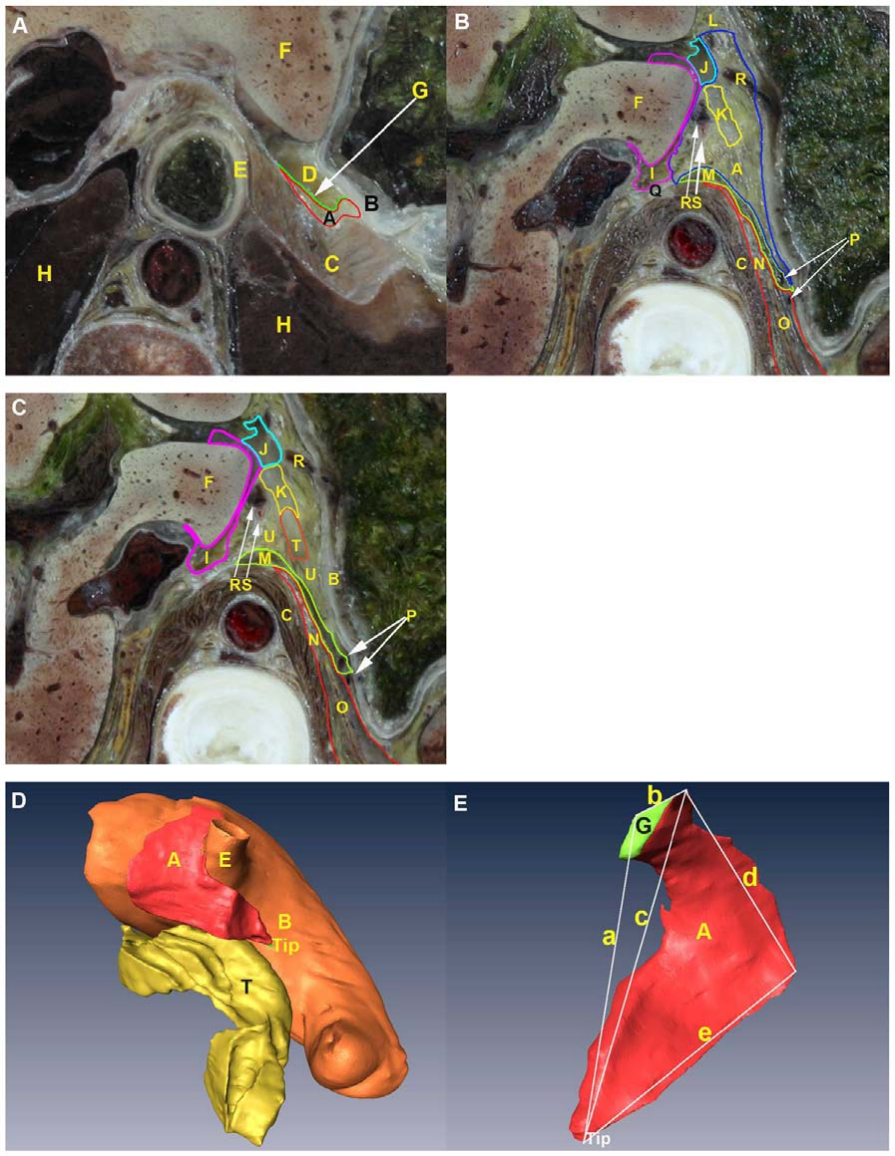
Visualization of the anatomy of the LES on CVH2. The LES is imaged on cross-sections from the inferior (A–C) and in 3D (D, E). (A) Starting cross-section of the LES. (B) Ending cross-section of the LES. On this section, the gastropancreatic ligament serves as the bottom margin of the LES. (C) Cross-section of the superior part of the pancreatic body. The appearance of the pancreatic body marks the disappearance of the LES; namely, the appearance of the peripancreatic space. (D) Posterointernal view of the spatial relationships of the LES to the stomach and pancreas. (E) Anteroexternal view of the LES. A: LES; B: gastric wall; C: left diaphragm; D: hepatogastric recess; E: distal esophagus; F: liver; G: upper segment of the left layer of the gastrophrenic ligament; H: lung; I: superior recess of the omental bursa; J: foramen bursae omenti majoris; K: inferior recess of the omental bursa; L: lesser omentum; M: gastropancreatic ligament; N: left retroperitoneal space; O: left adrenal gland; P: posterior gastric vein; Q: narrow space; R: left gastric vein and its branches; S: left gastric artery; T: pancreas; U: peripancreatic space; a: anterointernal leg; b: superior margin; c: diagonal line; d: posteroexternal leg; e: inferior margin.

**Figure 3:**
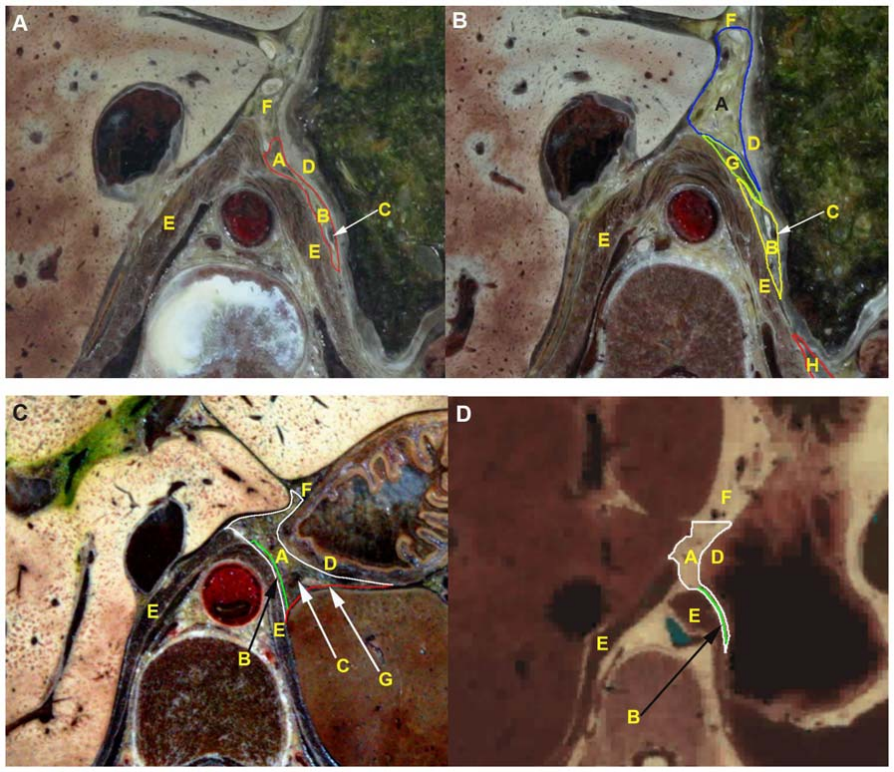
Spatial relationship between the GBA and LSFS on cross-sections from the inferior. (A) The GBA and LSFS overlap on upper sections of CVH2. (B) The GBA and the LSFS are separated by the lower segment of the left layer of the gastrophrenic ligament on lower sections of CVH2. (C, D) The LSFS is superimposed on the GBA on cross-sections of the CVH1 and VHMA. The thin adipose layer located between the white and green lines represents the LSFS. A: GBA; B: LSFS; C: posterior gastric vein; D: gastric wall; E: diaphragm; F: lesser omentum; G: lower segment of the left layer of the gastrophrenic ligament; H: left retroperitoneal space.

The LES is located between its anteroexternal surface and the diaphragm (Fig. 2A, B; Fig. 3). The location of the anteroexternal surface of the LES that adheres to the digestive tract varies greatly, contributing to the change in location of the LES, especially the tip (Fig. 2D). As shown on successive cross-sections of the CVH2 and VHMA, the anteroexternal surface of the LES adheres to the posterior wall of the abdominal portion of esophagus, the proximal portion of lesser curvature, the gastric cardia and fundus, and the upper portion of the gastric body. The tip of the LES extends downward excessively. These results were confirmed on the 3D model of CVH2 (Fig. 2D). In CVH1 and VHBM, the anteroexternal surface of the LES adheres to a similar area of the digestive tract, except for the upper portion of the gastric body.

#### Morphology of the anteroexternal surface

**Figure 4:**
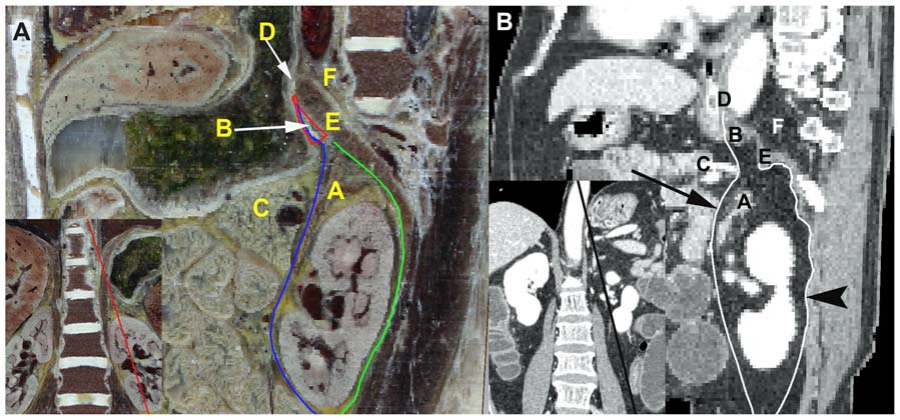
Manifestation of the spatial relationship between the LES and left retroperitoneal space on oblique sagittal planes. (A, B) Visualization of the communication relationship between the left retroperitoneal space and the LES from the lateral superior view on CVH2 and CT images, respectively. The anterior renal fascia is represented as a blue line and labeled by a black arrow on CVH2 and CT images, respectively. The posterior renal fascia is represented as a green line and labeled by an arrowhead on CVH2 and CT images, respectively. A: left adrenal gland; B: LES; C: pancreas; D: esophageal hiatus; E: left diaphragm; F: retrocrural space.

**Figure 5:**
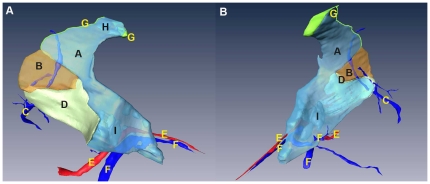
Visualizing the distribution of internal structures of the LES on CVH2 by transparent processing. (A, B) Posterointernal and anteroexternal view of the LES in 3D. The left gastric artery and vein pass through the anteroinferior part of the GBA and enter the lesser omentum. The left gastric lymph nodes are situated behind the left gastric vessels in the GBA, and the left cardiac lymph nodes are situated at the fat pad of the GBA. These structures are located at the corresponding position in the LES. The trunk of the posterior gastric vein passes through the lower segment of the left layer of the gastrophrenic ligament, the GBA, the LSFS, and the GBA, near the posterior border of the LES. Finally, this trunk penetrates through the upper segment of the left layer of the gastrophrenic ligament and enters the posterior wall of the gastric fundus. Similarly, the posterior branch of the posterior gastric vein goes straight through the lower segment of the left layer of the gastrophrenic ligament at its posterior margin and reaches the gastric wall. A: GBA; B: LSFS; C: posterior gastric vein and its branches; D: lower segment of the left layer of the gastrophrenic ligament; E: left gastric artery; F: left gastric vein and its branches; G: upper segment of the left layer of the gastrophrenic ligament; H: left cardiac lymph nodes; I: left gastric lymph nodes.

In the 3D model of CVH2, the anteroexternal surface of the LES appears trapezoidal in shape (Fig. 2E). The superior margin of the trapezoid corresponds to the upper border of the posterior wall of the abdominal portion of the esophagus. This superior margin attaches to the dome of the left diaphragm (Fig. 4). The inferior margin is identified by the gastropancreatic ligament (Fig. 2B, C). The anterointernal leg of the trapezoid is defined by the upper segment of the left layer of the gastrophrenic ligament and the lesser omentum (Fig. 2B, C; Fig. 3). The posteroexternal leg is delineated by the left layer of the gastrophrenic ligament (Fig. 5). The diagonal line derived from the anteroinferior tip of the trapezoid, the posteroexternal leg, and the inferior margin constitute a triangle (Fig. 2E). This triangle with the base facing the posterosuperior direction, and the tip facing the anteroinferior direction, excludes the adipose tissue at the cardiac notch of the stomach. The adipose tissue is overlain by the upper segment of the left layer of the gastrophrenic ligament (Fig. 2A, E).

### 2 Spatial relationships of the LES to the GBA, lesser omentum, and hepatic bare area

On cross-sections from the four Visible Human datasets and the 3D reconstructed models based on CVH2, the LES is comprised of the left sub-diaphragmatic fat space (LSFS) and the GBA. The GBA occupies most of the LES (Fig. 5). On successive cross-sections from the four samples, there were three different spatial relationships between the GBA and LSFS. The CVH2 and VHBM showed similar spatial relationships. On upper sections of these two samples, the GBA is located at the anterointernal portion of the LES, and the LSFS is situated at the posteroexternal portion of the LES. There is no distinct demarcation between the GBA and LSFS; they overlap with one another (Fig. 3A, Fig. 5A). On lower sections of the samples, the lower segment of the left layer of the gastrophrenic ligament serves as the left boundary of the GBA (Fig. 3B; Fig. 5). For CVH1 and VHMA, the LSFS lateral to the left crura of diaphragm is superimposed on the GBA on both upper and lower cross-sections (Fig. 3C, D). In addition, the anterointernal portion of the LES is tightly adjacent to the lesser omentum, and there is no obvious demarcation between them (Fig. 2B, Fig. 3). The right rear of the LES communicates with the hepatic bare area via a narrow space between the superior recess of omental bursa and the right crura of the diaphragm (Fig. 2B).

### 3 Distribution of internal structures of the LES

The appearance frequency of left gastric vessels and left gastric lymph nodes are 100% on the consecutive cross-sections of the four samples. The appearance frequency of posterior gastric vessels is 75%. The appearance frequency of the cardiac branch of the left gastric artery, inferior phrenic vessels, and left cardiac lymph nodes are 50%. Finally, the appearance frequency of right cardiac lymph nodes is 25%. Tab 1 gives the detailed distribution of internal structures of the LES in the four samples. Fig. 5 shows the specific distribution of internal structures of the LES for CVH2.

**Table 1:** Distribution of the internal structures of the LES.

	CVH1	CVH2	VHBM	VHMA
Left gastric vessels	Yes	Yes	Yes	Yes
Posterior gastric vessels	Yes	Yes	Yes	No
Cardiac branch from LGA	No	No	Yes	Yes
Inferior phrenic vessels	No	No	Yes	Yes
Left cardiac lymph nodes	No	Yes	Yes	No
Right cardiac lymph nodes	No	No	Yes	No
Left gastric lymph nodes	Yes	Yes	Yes	Yes

Yes = corresponding vessels were seen on cross-sections from Visible Humans datasets; No = corresponding vessels were not seen on cross-sections from Visible Humans datasets; LGA = left gastric artery.

### 4 Spatial relationship between the LES and left retroperitoneal space

**Figure 6:**
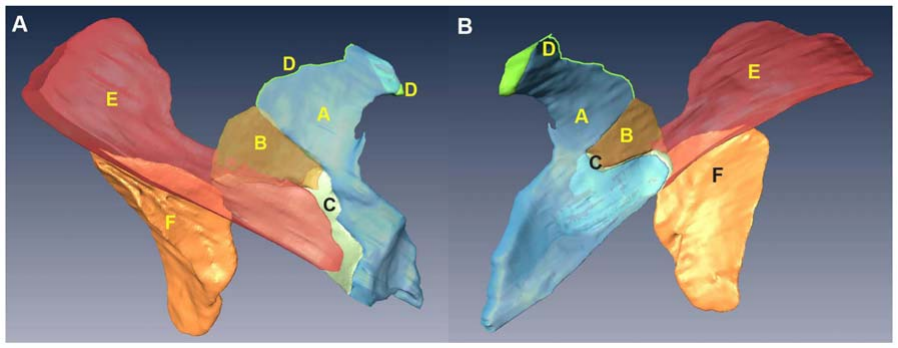
Visualizing the spatial relationship of the LES to the left retroperitoneal space on CVH2. (A, B) Posterointernal and anteroexternal view of the spatial relationship between LES and left retroperitoneal space in 3D. The upper pole of the left retroperitoneal space overlaps with the lower portion of the LSFS, but the left retroperitoneal space is separated from the GBA by the lower segment of left layer of the gastrophrenic ligament. The left retroperitoneal space communicates with the LES across the LSFS. A: GBA; B: LSFS; C: the lower segment of left layer of the gastrophrenic ligament; D: the upper segment of left layer of the gastrophrenic ligament; E: upper portion of the left retroperitoneal space; F: left adrenal gland.

As shown in Fig. 4, the upper pole of the left perirenal space is open to the LES. The anterior renal fascia spreads into the LES in the cranial direction and further extends to the dome of the diaphragm immediately posterior to the abdominal portion of the esophagus, and the superior portion of the left anterior pararenal space communicates with the LES in the cephalic direction. The communication relationship between the left retroperitoneal space and the LES in CVH2 is shown in Fig. 6.

### 5 LES on CVH2 images corresponding with that on MDCT images

**Figure 7:**
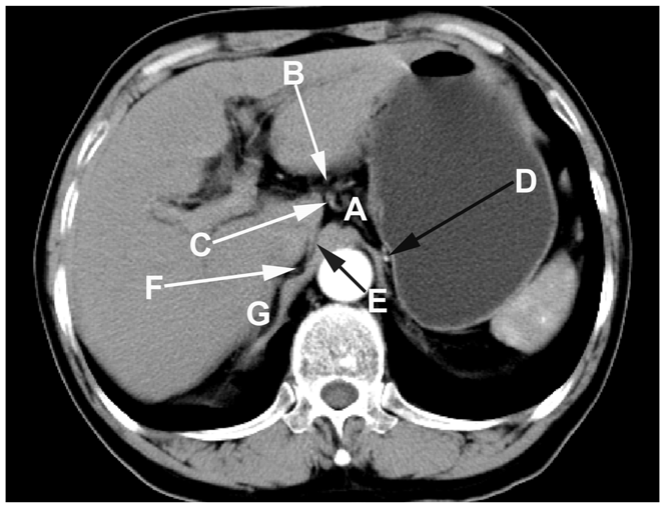
Manifestation of the LES on axial CT image from the inferior. A: LES; B: lesser omentum; C: left gastric artery; D: posterior gastric artery; E: narrow space; F: hepatic bare area; G: right adrenal gland.

**Figure 8:**
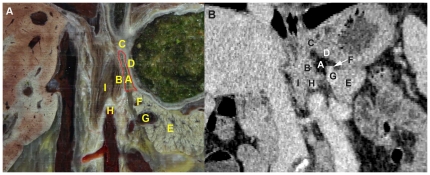
Presentation of the triangular-shaped LES on coronal MPR images from the front. (A, B) Visualization of the LES on CVH2 and CT images, respectively. A: LES; B: left crura of the diaphragm; C: gastric cardia; D: gastric fundus; E: pancreas; F: splenic artery; G: splenic vein; H: root of the celiac trunk; I: right crura of the diaphragm.

The anatomical location of the LES, its associated blood vessels, and its spatial relationships to the hepatic bare area and lesser omentum on CVH2 images (Fig. 2B) is well depicted on the axial CT images (Fig. 7). The anatomical location and morphology of the LES on CVH2 images is well depicted on the coronal CT images created with the MPR technique (Fig. 8).

## Discussion

Although much research concerning the communication relationships among sub-diaphragmatic spaces has been done by gross anatomy, thick-slice sectional anatomy, and CT images before, the thin-slice cross-sections retrieved from the CVHP and VHPA datasets are seldom used in such research. The cross-sections retrieved from Visible Human datasets provide images of a higher resolution than those obtained from CT or MRI scans, and with more detailed anatomical information regarding the human body than those from thick-slice sectional anatomy. This type of cross-sectional image, therefore, allows visualization of the spatial relationships of the LES to its adjacent spaces, and visualization of anatomical features of the LES in random sections and in 3D by using Amira® software.

In 1981, McCort et al. proposed that the left sub-diaphragmatic fat lay just to the inferior surface of the left diaphragm [24]. In our study, we show that parts of the left sub-diaphragmatic fat participate in composing the LES. This part of the left sub-diaphragmatic fat is defined as the LSFS in present research. As shown in Figs. 3 and 5, we confirm that the GBA and the LSFS comprise the LES. Various features in the Amira® software, such as transparency control, specific structure selection, animation, and a variety of manipulation modes, make visualization of the subtle spatial relationship of the GBA to the LSFS a simple exercise. The GBA and LSFS overlap on upper sections of the CVH2 and VHBM, and the LSFS that is lateral to the left crura of the diaphragm is superimposed on the GBA on successive cross-sections of the CVH1 and VHMA (Fig. 3). Therefore, there is no distinct boundary between the GBA and LSFS. We suggest that LES should replace GBA as terminology to define the extraperitoneal space that is located between the diaphragm and the posterior wall of the proximal portion of the gastric lesser curvature, and the gastric cardia and fundus on CT or MRI images.

Visualization of the LES and its spatial relationship to the GBA can help differentiate the GBA from the LES. Defining this extraperitoneal space with the correct anatomical terminology will be beneficial for academic communication in clinical practice and research. In addition, identification of the anatomical location of the LES will increase the diagnostic accuracy of LES abnormalities and the success rate of proximal gastric cancer surgeries that involve the posterior wall of stomach. Therefore, anatomists, clinicians, and radiologists should agree on an anatomical definition of the LES.

We argue that the reason why the anteroexternal surface of the LES is often viewed as an inverted triangle is that the connective and adipose tissue is relatively thin at the cardiac notch of the stomach in some people [12], [16]. The content of this area is mostly composed of adipose tissue, especially in people who are obese or have an excess of corticosteroids. This adipose tissue forms the “fat pad”, such as in CVH2 and VHMA [13]. The appearance of the fat pad contributes to forming the trapezoidal shape of the anteroexternal surface of the LES (Fig. 2E). In a retrospective study in 2005 of patients who had laparoscopic adjustable gastric-band surgery, Lyass and Parikh et al. argued that postoperative obstructions were attributable to an excessive, esophagogastric fat pad [25], [26]. Thereby, they suggested the routine excision of this fat pad during the operation.

Presentation of the course of the left gastric artery and vein within the LES in a 3D digital model is necessary for preoperative planning and simulation, for embolization or ligation of the left gastric artery pseudoaneurysm in acute pancreatitis and portoazygos devascularization in patients with portal hypertension [27], [28]. The incidence of vessels in the LES during gross anatomy examination was reported as 71.6% [29]. These vessels were composed of the left gastric vessels, the cardiac branch of the left gastric artery, the posterior gastric vessels, and the inferior phrenic vessels. Only the VHBM dataset presents all of these vessels in the LES on successive cross-sections. Our observation results to four samples on cross-sections are consistent with previous studies that were not prone to identify small and micro-vessels in the CVHP and VHPA [22]. We should improve the technique and materials for perfusion in the CVHP and VHPA [30], [31]. Furthermore, combining the vessel cast technique with modern imaging will better compensate for the unsatisfactory visualization of other vessels in the LES [32].

During the process of creating a proximal pouch of cardia in open Roux-en-Y Gastric Bypass surgery for curing obesity and type-II diabetes mellitus, the lesser omentum is opened in the LES by the surgeon [13]. Our study confirms this adjacent relationship between the lesser omentum and the anterointernal portion of the LES (Fig. 2B, Fig. 3).

As shown in Fig. 2B and Fig. 7, the LES communicates with the hepatic bare area via a narrow space between the superior recess of the omental bursa and the right crura of the diaphragm. The upper pole of the right perirenal space is open to the hepatic bare area and the LES may communicate with the right perirenal space across the hepatic bare area. As shown in Fig. 4, our findings agree with the communication relationships between the upper pole of the left perirenal space and the LES, and between the left anterior pararenal space and the LES [10], [33]–[35]. Our study demonstrates the communication relationship between the LES and the bilateral sides of the retroperitoneal space, which lays the foundation for further research into the dynamic pathways of peripancreatic fluid drainage into the mediastinum in acute pancreatitis [36].

In summary, this research presents a detailed spatial analysis of the LES and its related spaces via 3D visualization using data from the Visible Human Project. These relationships cannot be easily displayed using other imaging modalities. The controversial view of the morphology of the LES anteroexternal surface was clarified and better defined using this technique. Moreover, efforts to extend this technique to explore detailed communication relationships among other abdominal spaces are underway.

## Supporting Information

Movie S1 200 images are captured for presentation of a 3D model of the GBA and the LSFS with internal structures that rotate 360°.
(MPG)

Movie S2 175 images are captured for presentation of the LES with its adjacent stomach, left adrenal gland, and pancreas that rotate 360°.
(MPG)
